# The long-term health and wellbeing impacts of Healthy New Towns (HNTs): protocol for a baseline and feasibility study of HNT demonstrator sites in England

**DOI:** 10.1186/s40814-020-0550-2

**Published:** 2020-01-10

**Authors:** Paul Watts, Susanna Rance, Victoria McGowan, Heather Brown, Clare Bambra, Gail Findlay, Angela Harden

**Affiliations:** 1 School of Health, Sport and Bioscience, College of Applied Health and Communities, University of East London, Stratford Campus, Water Lane, London, E15 4LZ UK; 2 Institute for Health and Human Development, University of East London, Stratford Campus, Water Lane, London, E15 4LZ UK; 3 Institute of Health and Society, Newcastle University, Sir James Spence Institute, Royal Victoria Infirmary, Newcastle-upon-Tyne, NE1 4LP UK; 4 Fuse—UKCRC Centre for Translational Research in Public Health, Upon Tyne, Newcastle, UK; 5 Barts Health NHS Trust, London, UK

## Abstract

**Background:**

Increasing levels of non-communicable diseases (NCDs), mental health problems, high rates of unhealthy behaviours and health inequalities remain major public health challenges worldwide. In the context of increasing urbanisation, there is an urgent need to understand how evidence that living environments shape health, wellbeing and behaviour can be used to design and deliver healthy environments in local urban settings. The Healthy New Town (HNT) programme implemented in England from 2015 consists of ten major housing developments that aim to improve population health through healthy design principles, new models for integrating health and social care and the creation of strong and connected communities. The programme provides a natural experiment in which to investigate the effects on health, wellbeing and inequalities of large-scale interventions targeting the wider social determinants of health.

**Methods:**

The research described in this protocol aims to examine the feasibility of a larger study to assess the longer-term health impacts of HNTs, by addressing two research questions: (1) what are the similarities and differences in the HNT programme developments, processes, contexts and expected impacts and outcomes across HNT sites? and (2) how feasible is the use of data from routine sources and existing HNT evaluations and as the baseline for a definitive study to assess impact on health, wellbeing, behavioural and economic outcomes and programme processes? The research will consist of (a) participatory systems mapping with stakeholders to produce a theoretical framework for a longer-term study on the HNT programme, (b) synthesis of existing qualitative data from local HNT evaluations to understand local processes and intervention mechanisms, (c) scoping local and routinely available data to establish a baseline and feasibility for a longer-term study of health and economic outcomes, and (d) building relationships and recruiting HNT sites into the proposed research.

**Discussion:**

The proposed research will produce a theoretical framework and assess the feasibility of a definitive study of outcomes of the HNT programme. This research is necessary to understand how longer-term health, wellbeing, behavioural and economic outcomes can be measured, and to inform a definitive study to generate evidence on the effectiveness of the HNT programme.

## Background

Improving health and reducing health inequalities remain major global public health challenges. Increasing levels of non-communicable diseases (NCDs), mental health problems, rising levels of obesity and high rates of physical inactivity, unhealthy diets, smoking and harmful alcohol consumption are placing increasing pressure on health services [1, 2]. The costs associated with smoking, physical inactivity and overweight and obesity, for example, are estimated to be around £12.6 billion annually in England and Wales in the UK [3]. These problems are compounded by the clustering of NCDs, mental health problems and health risk behaviours in the most disadvantaged groups [4, 5], as well as multi-morbidity within a population that is getting older [6]. There is an urgent need to find effective approaches to prevention, particularly upstream interventions which impact on the social determinants of health [7].

Health and health behaviour are influenced by interactions between characteristics of individuals and their places of residence [8–10]. Changes to specific components of local environments can improve physical and mental wellbeing, promote healthy behaviours and reduce health inequalities [11–13]. Embedding healthy principles in town and city planning is endorsed in WHO recommendations to ‘place health and health equity at the heart of governance and planning’ [10]. However, the processes and longer-term impacts of creating healthy urban environments are less well understood [14]. Developing an understanding of how characteristics of urban environments interact as whole systems to affect health and behavioural outcomes is a pressing priority [14].

The Healthy New Town (HNT) programme was funded by the English National Health Service in 2016 for 3 years. The programme brings together partners in local government, housebuilding, local communities and healthcare to offer improved choices and opportunities for healthy living through healthy placemaking. The HNT programme draws upon evidence from research on characteristics of healthy living environments [15] and recommendations from leading global agencies, including the WHO recommendations on health and health equity for governance and planning [10], and United Nations recommendations for safe, inclusive, resilient and sustainable cities to promote healthy lives and wellbeing [16]. Previous programmes and research studies, including those related to the European Healthy Cities movement [17] have drawn upon this evidence base and associated recommendations with a focus on very large metropolitan areas [15]. The scale of the sites in the HNT programme differs from these programmes and research studies as it focuses on developments within smaller towns or urban areas.

HNTs are 10 major housing developments across England, referred to by NHS England as ‘HNT demonstrator sites’. These HNT demonstrator sites were selected from an initial 114 expressions of interest in 2016. Sites were selected to represent a diverse cross-section of housing developments planned across England, including those from high-volume builders and housing associations, across a wide range of land values [18]. Sites were also selected to represent diversity in sociodemographic characteristics and health needs. NHS England has announced that there are plans for over 76,000 new homes across the ten HNT sites, with potential total capacity for approximately 170,000 residents [18]. The size and nature of the planned developments and the timescales for their completion vary considerably. For example, the planned development of 885 houses to be built by 2023 at the HNT site in Barton, Oxfordshire is relatively small compared to the development of 15,000 houses planned to be built in Ebbsfleet, Kent by 2035 [18]. Further details of the new housing developments planned at each HNT demonstrator site are provided below under the ‘Settings and interventions’ section.

HNTs are united by a shared aim to improve population health and reduce inequalities by applying healthy design principles that cover movement and transport, green and social infrastructure, the local economy, food choices and placemaking [19]. The three HNT programme priorities are: (1) planning and designing a healthy built environment, (2) creating innovative models of healthcare, and (3) encouraging strong and connected communities [20]. These broad programme priorities are common to the 10 HNT demonstrator sites. However, the diversity of local circumstances dictates that each site will have a differing list of specific priorities and different perspectives on the most suitable type of interventions and services that can be delivered to achieve these priorities. Examples of specific interventions are provided below under the ‘Settings and interventions’ section.

At the outset of the HNT programme there was no formal plan or funding arrangement in place to evaluate the longer-term impact of the HNT programme on health, wellbeing and economic outcomes. However, some HNT sites had commissioned academic partners to evaluate the impacts of specific interventions and processes of the HNT programme. Five HNT sites engaged in a formative evaluation of the programme which ended in March 2019. Site leads, funders and evaluation partners at these sites—Barking Riverside, Bicester, Darlington, Ebbsfleet and Whitehill & Bordon—form the HNT Evaluation Collaborative (HNTEC). The HNTEC was formed to facilitate learning across sites and to explore opportunities for further evaluation, including evaluation of the longer-term impacts of the HNT programme on health and wellbeing outcomes. As academic partners of the Evaluation Collaborative, a team of researchers from the University of East London and Newcastle University (the authors) sought and received funding from the National Institute of Health Research to investigate the feasibility of a study of the longer-term impacts of the HNT programme. The research team is working collaboratively with the HNTEC, but we do not have a role in the planning, design or implementation of the HNT programme. This protocol describes the feasibility research which was planned with a focus on the 5 HNT sites within the HNTEC.

The HNT programme provides a natural experiment in which to investigate the effects on health and wellbeing of large-scale, whole system interventions. Research on interventions addressing the social determinants of health requires methods that recognise the complexity of pathways towards impact [21]. Natural experiments can provide valuable evidence on the impact of large-scale interventions [22], and a whole systems approach can investigate pathways to health improvement over the longer term [23]. Previous evaluations of large-scale, area-based interventions such as New Deal for Communities [24] and the Healthy Communities Challenge Fund [25], describe the importance of setting realistic targets for similar schemes based on their period of funding. HNT sites have broad programme objectives which are not measurable within the 3 years of current funding allocated. For example, reducing the gap in healthy life expectancy could take over 10 years to be demonstrated.

A robust evaluation should ask: what happens when an intervention is ‘implemented across a range of contexts, populations and subpopulations?’ [26] And, where there is evidence that an intervention has had an effect on health outcomes, the evaluation should ask ‘how have these effects come about?’ [26]. The HNT programme offers a novel opportunity to adopt a whole systems approach to understanding the complexities of implementing a programme of area-based interventions targeting the social determinants of health in real world, dynamic settings across several sites in England.

### Aim

The research described in this protocol aims to examine the feasibility of a larger study to assess the long-term health impacts of HNTs which can then inform the future planning, development and implementation of healthy urban environments.

### Research questions

To determine the feasibility of a larger study to assess long-term health impacts of HNTs, it is first necessary to understand more about the developments and interventions taking place at each of the HNT sites. This includes understanding the ways in which each HNT site has interpreted HNT priorities and plans to act upon them in the context of their site-specific priorities. The research outlined in this protocol therefore aims to answer the following research question:

1. What are the similarities and differences in HNT programme developments, activities and processes, contexts and expected impacts and outcomes across the 5 HNT sites that form the HNT Evaluation Collaborative?

The feasibility of a future study on the longer-term impacts of the HNT programme is dependent on the availability of quantitative data, either held locally at HNT sites or from routinely available sources, to form a baseline against which any changes in health outcomes can be assessed. Similarly, the feasibility of a longer-term process evaluation of the HNT programme is dependent on the availability of qualitative and quantitative data on the processes and mechanisms through which HNT programme activities and interventions operate. Therefore, the second research question to be addressed by the research detailed in this protocol is as follows:

2. How feasible is the use of data from routine sources and existing HNT evaluation data as the baseline for a longer-term study to assess impact on health, wellbeing, economic, and behavioural outcomes; programme processes; and mechanisms?

### Objectives

To answer these research questions, the research described in this protocol will need to achieve the following objectives:

1. To produce systems maps for a sample of sites within the HNT Evaluation Collaborative, illustrating processes and expected outcomes from HNT activities and developing a theoretical framework for longer-term research. These systems maps will contribute to a more detailed and holistic understanding of the processes and mechanisms through which HNT programme activities and interventions operate, and the outcomes that the HNT programme is expected to influence.
2. To synthesise any available qualitative data produced through existing local evaluations and to test the feasibility of using this data to ground a longer-term qualitative study of HNT processes. Synthesis of this data will help to provide an understanding of common themes relating to the ways in which processes and interventions within the HNT programme have been experienced by residents and other stakeholders.
3. To define primary and secondary outcome measures for assessment of HNT effectiveness and cost-effectiveness using existing HNT datasets and routinely available data. The HNT programme has broad shared priorities, but a longer-term study of effectiveness and cost-effectiveness will require clearly defined outcome measures and will depend on the availability of data to operationalise these measures.
4. To test the feasibility of including all 10 HNT demonstrator sites in the study to improve study power and representativeness. A holistic study of the impacts of the HNT programme should include all 10 HNT demonstrator sites. It is therefore important to investigate the feasibility of including HNT sites that are not part of the HNTEC and may not currently be seeking to evaluate the health impacts of developments and activities at these sites.
5. To set up an HNT Residents’ Group and wider Stakeholder Groups to advise the study, incorporating their input into the baseline study and longer-term research design. Patient and public involvement (PPI) will form an essential part of a potential longer-term study. It is therefore necessary, as part of the proposed feasibility research, to consolidate existing relationships with residents and support residents to work together with other stakeholders and the researchers to inform the research from design through to dissemination.

## Overview

**Fig. 1:**
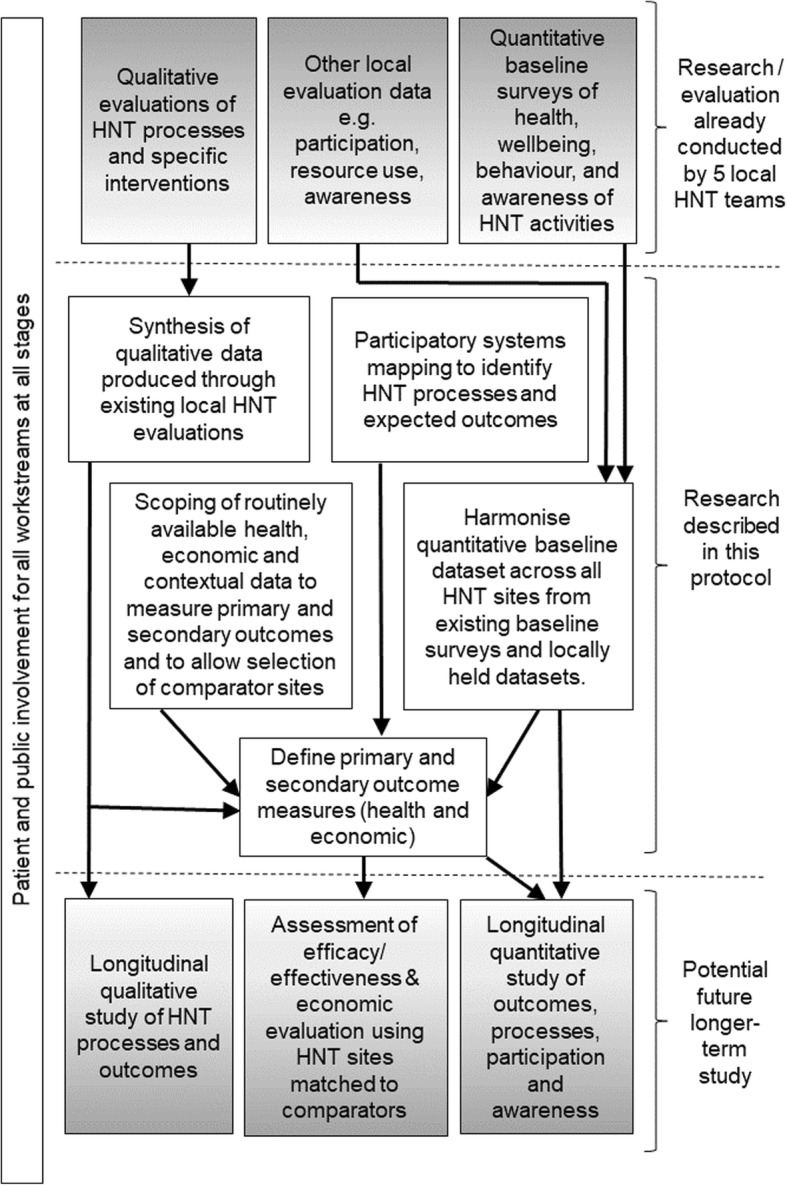
Overview of research processes

The proposed research involves harmonising and synthesising existing data from local HNT evaluations, defining appropriate health, wellbeing and economic outcomes and testing the feasibility of future research over a longer period for all HNT sites. Our research design is informed by a whole systems approach using participatory mapping in a sample of HNT sites with stakeholders including residents [27]. Researchers will elicit key variables to develop a qualitative conceptualisation of the system and its boundaries, using a framework that understands the programme within complex and adaptive social systems [28]. Development of the HNT system will provide a road map for longer-term research by identifying key change elements to be monitored over time. An overview of all research processes is illustrated in Fig. 1.

### Establishing a research partnership with HNT stakeholders

Five HNT sites (Barking Riverside, Bicester, Darlington, Ebbsfleet and Whitehill & Bordon) form the HNT Evaluation Collaborative (HNTEC). Some of these sites have conducted baseline surveys and, using varied approaches, have conducted focus groups on resident experiences of HNT developments, processes and specific interventions. In Darlington HNT, participatory systems mapping workshops have developed a theoretical framework that demonstrates how HNTs might trigger feedback loops that influence systems operating on the sites. We will test the feasibility of expanding the research programme from 5 to all 10 HNT sites. We aim to develop relationships with the remaining 5 sites (Cranbrook, Whyndyke Farm, Halton Lea, Northstowe and Barton Park), and if feasible to recruit them to participate in the research processes described below and recruit them to join a future longer-term study.

### Settings and interventions

**Fig. 2:**
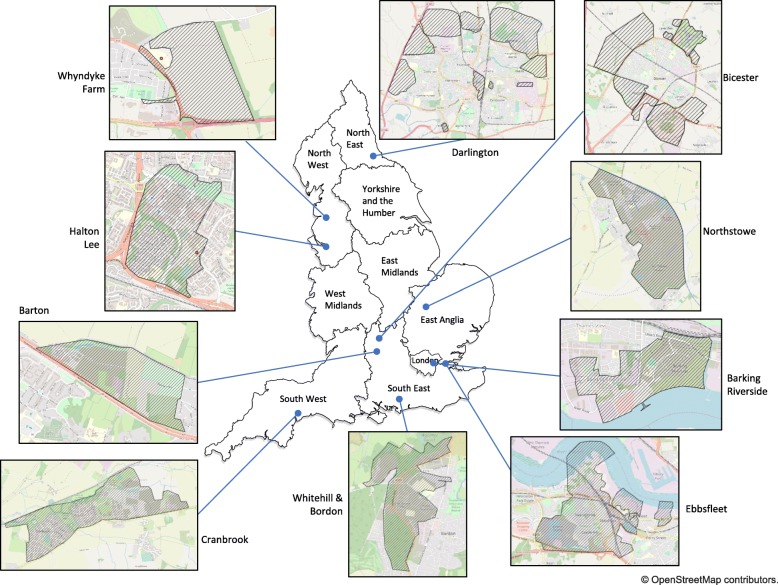
Map of locations of HNT sites and developments

The initial setting for this baseline and feasibility study consists of 5 of the 10 HNT sites that are part of the HNT Evaluation Collaborative (with an aim of recruiting additional sites, as described above). Sites encompass not only the new housing developments but also surrounding areas. The geographical sizes of HNT sites and developments vary. Sites can be defined approximately using Middle Layer Super Output Areas (MSOAs). Most are made up of two MSOAs (each with a minimum population size of 5000; mean of 7200). It will be necessary, through the feasibility research described in this protocol, to investigate how the geographical boundaries of HNT sites can be defined accurately and consistently. For example, if the definition of an HNT site includes areas surrounding housing developments, there is a need to identify how these surrounding areas can be defined consistently with stakeholder agreement regarding the accuracy of such definitions. Details of the new housing developments planned at each HNT demonstrator site are shown in Table 1. The geographic location of the HNT sites and locations of the developments at each site are shown in Fig. 2.

**Table 1:** New housing developments planned at the 10 HNT sites [19, 29]

HNT Site	Region	Number of homes planned	Planned year of completion	Land usage
1. Barking Riverside, London*	London	10,800	2031	Brownfield
2. Barton, Oxford	South East	885	2023	Greenfield
3. Bicester, Oxfordshire*	South East	13,000	2038	Greenfield
4. Cranbrook, Devon	South West	8000	2028	Greenfield
5. Darlington, County Durham*	North East	3600	2025	Mixed
6. Ebbsfleet Garden City, Kent*	South East	15,000	2035	Brownfield
7. Halton Lea, Runcorn	North West	800	2028	Brownfield
8. Northstowe, Cambridgeshire	East Anglia	10,000	2028	Brownfield
9. Whitehill & Bordon, Hampshire*	South East	3350	2036	Brownfield
10. Whyndyke Garden Village, Lancashire	North West	1400	2031	Greenfield

*Sites forming the HNT Evaluation Collaborative.

The HNT sites aim to improve population health and reduce inequalities by embedding 10 healthy design principles which draw upon national and international evidence and guidelines on healthy planning and design [10, 16]. The description of the 10 healthy design principles below has been adapted from NHS England’s ‘Putting Health into Place’ publication [20].

1. *Plan ahead collectively*. An authentically ‘whole systems’ approach to creating healthier places requires meaningful collaboration between a diverse mix of people and organisations with a shared vision and clear objectives. To be able to ‘plan ahead collectively’ HNTs will need to form partnerships with representatives from local councils, public health, health providers, housing developers and housing associations, community organisations, residents and businesses.
2. *Plan integrated health services that meet local needs*. An understanding of the specific local health needs of the populations at each HNT site, and how these needs may change, is essential for the effective delivery of health services. HNT sites will need to plan and forecast health and care workforce requirements supported by the modern digital and technological infrastructure needed to deliver and manage health and care services.
3. *Connect*, *involve and empower people and communities*. Social relationships are essential for health and wellbeing. Strong communities that promote cohesion, inclusion and collective decision-making can help form a greater sense of connection with the places where people live and work. HNTs will need to use social and traditional media, community events and community leaders to create cohesive communities that make the most of collective skills and knowledge.
4. *Create compact neighbourhoods*. Neighbourhoods that are compact and well-connected tend to be more walkable and to promote physical activity and social interaction. HNTs will need to create neighbourhoods with an accessible mix of land uses (i.e. shops, jobs, services, home and schools), well-connected streets, cycling infrastructure, a culture of active travel and social interaction and a reduced reliance on cars.
5. *Maximise active travel*. Physical activity is vital for promoting better physical and mental health. HNT sites will need to plan neighbourhoods that help to promote active travel. Well-planned neighbourhoods will make active travel an appealing choice for all residents by providing appropriate infrastructure and affordable options for active travel. These may include well-designed and maintained walking and cycling paths with appropriate signage and the use of digital technology to provide information, help plan journeys and promote use.
6. *Inspire and enable healthy eating*. Making it easier and more affordable for people to follow healthier diets is key to reducing health inequalities. HNT sites will need to create places that facilitate access to healthy cooking ingredients and meals in the local area. As well as improving access to healthier foods, limiting access to less healthy foods may be possible through local authority planning and licencing decisions.
7. *Foster health in homes and buildings*. Workplace, school and residential buildings can have an important impact on health through the impact of their design on ventilation, lighting and privacy. Public buildings can also influence wellbeing by providing opportunities for social interaction or quiet reflection. HNT sites will need to use their influence on planning decisions to create homes and buildings that use innovative and technological solutions to create healthy buildings.
8. *Enable healthy play and leisure*. The availability of opportunities to make healthy choices about how to use leisure time can have an important influence on health and wellbeing. HNTs will need to provide opportunities for people of all ages to spend their leisure time being active, socialising and enjoying themselves. This can include providing safe spaces for social and physical activity suitable for all in local parks. These opportunities can be advertised and augmented using digital technology and social media.
9. *Provide health services that help people stay well*. The provision of support to allow people to stay healthy and to manage long-term conditions is central to new models of healthcare. HNT sites will need to strengthen primary care services and work with community organisations to provide appropriate out-of-hospital care services including peer support, health coaching, social prescribing and mobile apps to support the health and care needs of residents.
10. *Create integrated health services*. Planning the location and integration of a range of health services to improve support, diagnosis, treatment and care can improve the efficiency and effectiveness of health and care systems. HNT sites will need to enable staff and services including GPs, acute care services and mental health services to work together efficiently. This may include planning services to be located at the same site and using digital technology to help services work together more efficiently.

**Fig. 3:**
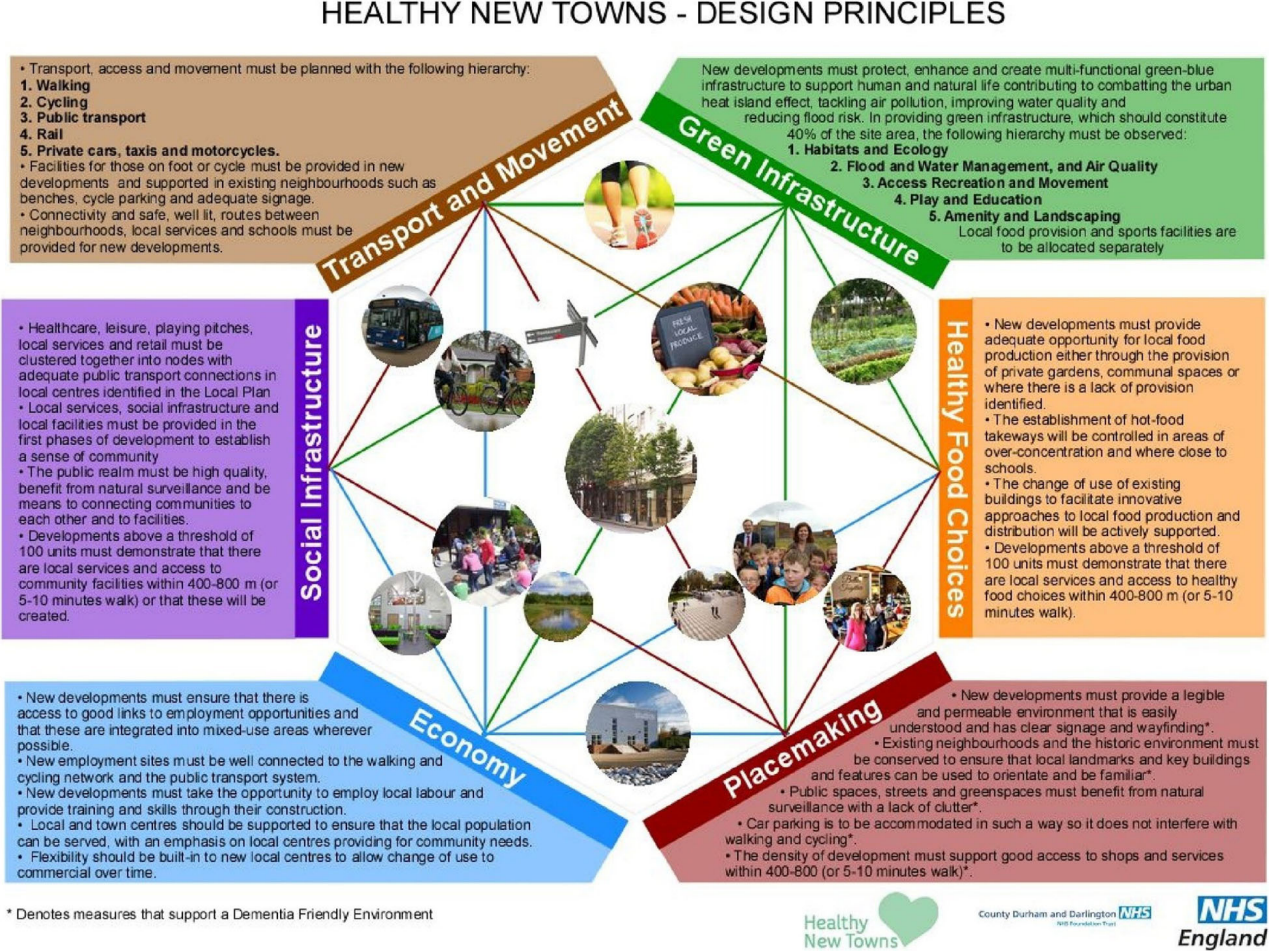
Darlington Healthy New Towns design principles

These broad design principles are shared across HNT sites, but the ways in which they are interpreted, integrated into development plans and applied in practice will vary between HNT sites depending on stakeholder perspectives and site-specific priorities. Figure 3 shows an example of how elements of these healthy design principles have been adapted and integrated into local HNT planning through Darlington HNT’s Design Principles Evidence and Practice Guide [19]. Some examples of specific interventions to be tested at HNT sites include fast food-free zones near schools in Barking Riverside (design principle 6); a free, rewards-based ‘Get Active’ smartphone app for new residents in Ebbsfleet (design principle 5); designing safe and appealing green spaces (design principles 3, 4, 5 and 8); building dementia-friendly streets (design principles 3, 4 and 8); and enhancing people's access to new GP services using digital technology (design principles 1, 2 and 10). Further examples and case studies are available in NHS England’s ‘Putting Health into Place’ publication [20]. An important part of the feasibility research proposed here will be to understand similarities and differences in the way these healthy design principles are applied and integrated into planning across the HNT sites.

### Participatory systems mapping

The overall aims of the HNT programme—to improve health, wellbeing and inequalities using a whole system approach—are very broad in scope. The feasibility of a longer-term study to assess whether these aims have been achieved will require clear definitions of expected outcomes and the processes through which these outcomes are expected to be achieved. Participatory systems mapping [27] is a method that can be used to develop a theoretical framework to illustrate how expected changes may occur. Participatory systems mapping [27] will be used to produce a framework for understanding variation, as well as similarity, in the local and overall effects of the programme in the different ‘systems’ in which the HNT programme is seeking to affect change. The resulting systems maps will contribute to an understanding of the processes and mechanisms through which HNT programme activities and interventions operate.

#### Participants, sampling and recruitment

The research team will run participatory systems mapping workshops with relevant stakeholders—including residents, decision-makers, planners, local politicians, voluntary sector representatives and academics—in the participating HNT sites that form the HNTEC. Participant numbers will vary per site depending on local programme size, but are expected to be between 12 and 20 per workshop, to incorporate representatives of different interest groups in the HNT and surrounding area and to provide an appropriate balance of stakeholder diversity.

#### Data collection

Qualitative data will be generated in 2-h local workshops (one per participating HNT site) incorporating a participatory systems mapping exercise. This exercise will map the different elements within the local HNT system and will require stakeholders to discuss their experiences and understandings of how the HNT programme and its processes operate and how specific outcomes may be influenced. Discussion topics will include the issues they were seeking to address through the HNT programme, the interventions they have implemented, their assumptions about how these interventions would affect change, any unintended consequences, and interactions between different elements in this system. In the first instance, systems mapping data will be collected across five HNT sites. We will generate data on the types of interventions implemented in HNT programmes, the contexts in which HNTs have been implemented, assumptions on how the programme will affect change and intended and unintended consequences. Participatory systems mapping methodology will be used to co-develop with the stakeholders a visual system and causal loop diagram in each HNT site demonstrating the different elements within the system and the links between them. Systems will be compared and integrated across sites (cases) to develop hypotheses for testing the impact of HNTs over the longer term.

#### Data analysis

The participatory mapping exercise will be used to produce a visual ‘system’ graphically illustrating the expected outcome of the HNT programme and the anticipated process through which outcomes will be achieved. The visual system will illustrate the ways in which specific interventions and actions within the HNT programme are expected to influence specific health and wellbeing outcomes. Researchers will use this visual system to develop a causal loop diagram (CLD) in VENSIM computer software. A CLD will demonstrate the different elements within the system, the links between them, how they are interconnected and how alterations to these elements may affect change. We will analyse ‘factors of influence’ [24] to identify potential causal factors for particular outcomes, considering criteria for establishing the sufficiency of these factors to explain the outcome. These CLDs will make explicit the assumptions expressed by participating stakeholders about the expected outcomes of the HNT programme and the mechanisms through which the outcomes are expected to be influenced. The resulting CLDs for each site will be compared in order to develop a national level ‘system’ which will be used to identify the elements in which the HNT is affecting change and develop hypothesised pathways to health improvement which can be followed longitudinally.

### Scoping and synthesis of qualitative data from existing local evaluations

Some HNT sites, including the 5 sites within the HNTEC, have conducted formative qualitative resident evaluations to understand experiences of HNT developments, processes and specific interventions. These evaluations have used different approaches and cover a broad range of topics and interventions. Therefore, synthesis of data from these evaluations will help to provide an understanding of common themes relating to the ways in which processes and interventions within the HNT programme have been experienced by residents and other stakeholders.

Synthesis of any available qualitative data from existing local evaluations will be brought together with findings from the participatory systems mapping to form the basis for a longer-term qualitative study of the processes and mechanisms through which the HNT programme operates. Some sites have evaluated HNT processes through interviews and focus groups with residents. Where individual sites have commissioned evaluation partners to carry out qualitative evaluations of specific interventions (e.g. a cycle training programme for residents), we will seek to collate and assess the relevance of these findings for the longer-term study.

#### Data collection

We will request access to any qualitative findings already produced by local evaluation teams at HNT sites. Local qualitative evaluations will be included in the synthesis if findings are publicly available or access to reports of findings are granted by local site leads and evaluators and there is ethical approval for findings to be disseminated in anonymised formats. Reports will be collated and assessed for relevance and quality, and findings extracted using a standardised table to be populated with key information about the qualitative evaluations (e.g. aims, participants, data collection and analysis).

#### Data analysis

Findings from local evaluations will be analysed to understand common themes relating to successes and challenges in implementing HNT principles. Existing qualitative data from local evaluations will be synthesised using thematic analysis [30] and fed into the local and national level system maps described above. We will map characteristics of participants in these evaluations and write a commentary on their diversity, as recorded in study documents, in terms of interest groups and protected characteristics.

### Harmonisation and scoping of quantitative data

The feasibility of a longer-term study of the effectiveness and cost-effectiveness of the HNT programme will not only require clearly defined outcome measures but also depend on the availability of data to operationalise these measures. We will scope the suitability of routinely available social, economic, demographic, health and environment data as (1) primary and secondary outcome measures, (2) a way to select comparator sites, and (3) parameters for the economic evaluation. This data may include, for example, data held by Public Health England (PHE) reporting on rates of chronic diseases such as cardiovascular disease and diabetes; data held by the Office for National Statistics on demographic characteristics, geographic characteristics such as land use and levels of active transport; and other anonymised data held by Local Authorities and Primary Care including local population health surveys, data on hospital admissions for specific health problems and General Practice prescription rates. We will also scope the availability of existing locally held quantitative datasets including resident surveys and other local evaluations using quantitative measures. If access is granted by local site leads/evaluators, there is ethical approval for information to be shared, and the measures can be matched across sites, we will harmonise data across HNT sites into a single dataset.

#### Data collection

A wide range of potential sources of routine data will be identified and assessed for potential relevance by the research team in collaboration with the HNT Evaluation Collaborative. Where individual sites have commissioned evaluation partners to carry out quantitative surveys of health and wellbeing, health behaviours, use of local resources, and awareness of local activities, we will seek to collate these into a national level dataset. An example of relevant data is a survey completed by a sample of 1106 Bicester HNT residents from April to June 2017. Any existing quantitative datasets collected as part of local evaluations will be identified via evaluation partners and site leads. For routine data and existing locally held quantitative data, we will collect metadata on the methods used to operationalise health, economic and behavioural measures approaches to sampling, and timing of data collection.

#### Data analysis

The analysis of this data will entail assessment of the feasibility of accessing the data required for a longer-term study of the effectiveness and cost-effectiveness of the HNT programme. For routinely available data we will use metadata to assess whether the available data are suitable for assessment of expected outcomes, available at suitable time points to retrospectively form pre-intervention baseline measures, and likely to be available at future intervals suitable for follow up. When primary and secondary outcomes have been defined and validated with key stakeholders, sample size and power calculations will be used to test the feasibility of using these data to form a baseline for a longer-term study. Using existing surveys and locally held datasets (e.g. residents’ surveys and local quantitative evaluations), we will test the feasibility of harmonising a quantitative baseline dataset across HNT sites. Where feasible, we will combine existing data into a single dataset, establishing where variables are comparable across sites. We will integrate survey data with other locally held quantitative datasets containing information on residents’ health, wellbeing and health behaviours. We will assess all data for quality (i.e. validity, reliability, timeliness, precision, integrity). Metadata will be used to assess comparability of measures across HNT sites and suitability for assessment of expected outcomes, whether these samples are representative of the HNT resident populations, and whether the timing of data collection is consistent across HNT sites, and therefore appropriate to form a pre-intervention baseline.

#### Control/comparator group

To be able to generate robust evidence on the effectiveness and cost-effectiveness of the HNT programme, it will be necessary to select control areas to compare the rate of change in outcome measures at the HNT sites with the rate of change in areas that are not part of the HNT programme. We will test the feasibility of using area-level propensity score matching [31] to match HNT sites to comparator sites [32]. The set of variables for which routinely available data is available will be narrowed through a Delphi panel composed of members of the Evaluation Collaborative and stakeholders including Residents’ Group members. Using methods described by de Vocht et al. [32], panel members will use experiential and expert knowledge to reduce the dataset to a narrower set of variables considered to be important influences on outcomes. We will assess the feasibility of using this approach to selecting variables as well as other appropriate approaches such as principle component analysis.

### Outcome measures

Primary and secondary outcomes will be selected based on findings from participatory systems mapping and availability of data from existing surveys/datasets from HNT sites and routinely available measures. Systems maps will guide the selection of outcomes to best match the changes theorised in the maps. These may include changes in health behaviour and levels of inequality, anticipated through changes to resources and the built environment. Selection of primary and secondary outcomes will also be informed by the HNT programme priorities and through stakeholder discussion.

**Table:** 

HNT Priority	Examples of potential outcomes	Examples of potential data sources
1. Planning and designing a healthy built environment	Physical activity levels, active travel, healthy eating, mental wellbeing, anxiety, happiness	Understanding Society, Annual Population Survey, Hospital Episode Statistics
2. Creating innovative models of healthcare	Health service utilisation, prescribing	Hospital Episode Statistics, NHS Digital
3. Encouraging strong and connected communities	Crime rates, anti-social behaviour, mental wellbeing, community cohesion, social capital	Annual Population Survey, Crime Survey for England and Wales, Home Office

### Health economic evaluation

The aim of the health economic analysis is to determine if HNTs are cost-effective in the long-term. To reach this aim we will scope existing data sources such as Hospital Episodes Statistics [33], Health Survey for England [34], Understanding Society Survey [35], English Longitudinal Survey for Aging [36], data held by local authorities and PHE [37] to identify appropriate economic outcomes for an economic evaluation. Part of this scoping exercise will determine if appropriate data is available at key time points to be used in an economic evaluation and if there are any gaps in the data which can be filled by primary data collection. In a definitive longer-term study, this data would be utilised in multivariate regression analysis to determine if the benefits of HNTs outweighed the costs of the programme.

### Criteria for progression to a longer-term study

We will progress to development and submission of a full proposal for a larger study of longer-term processes and outcomes if the following progression criteria are met: (1) Primary and secondary outcomes for the overall HNT programme can be defined based on the HNT programme priorities and findings from the participatory systems mapping. (2) A core set of these outcomes can be measured using routinely available or locally held data to retrospectively form a baseline, and these data are of acceptable quality (i.e. validity, reliability, timeliness, precision, integrity). (3) HNT sites can be matched to comparator sites retrospectively using such data. (4) Sample size and power calculations indicate that the data will be feasible to use as a baseline against which changes in outcomes can be measured. (5) Existing qualitative data from local HNT evaluations together with the findings of the participatory systems mapping can be used to inform a longer-term qualitative study on HNT processes and mechanisms. (6) Relationships with key stakeholders in HNTs can be developed and maintained beyond the initial formative evaluation period which ends in March 2019.

### Patient and public involvement

Throughout this research, we will embed best practice methods of public and patient involvement (PPI) and community and stakeholder engagement, using co-production approaches [38, 39]. We will actively engage and support residents from the HNT communities so that they can work together with other stakeholders and the researchers to inform the project at all stages, from design through to dissemination. At the heart of the PPI work, residents’ involvement will be facilitated and supported through establishment of an HNT Residents’ Group (RG). Two residents will be recruited from each of the HNT sites in the study, using maximum variation criteria to ensure diversity and inclusion from an equalities perspective. The RG will advise researchers on how best to involve community members from each site to ensure the research draws a wide range of residents into co-production processes. The RG will also be involved in the development of research and ethics materials, press releases, report drafts and dissemination. RG members will receive training, specific support before each meeting or workshop and recompense for their time dedication through vouchers following INVOLVE guidelines: http://www.invo.org.uk/find-out-more/what-is-public-involvement-in-research-2/. All research activities will incorporate elements of PPI, wider stakeholder engagement and co-production.

### Project management and governance

We will set up a Stakeholder Advisory Group building on the HNT Evaluation Collaborative (HNTEC) and expanding to include representatives of local Directors of Public Health, Councillors and Health and Wellbeing Boards, Residents’ Group, NHSE and PHE. This group will enable us to continue and extend the co-production approach we have taken, through our work with the HNTEC, in the conception, scoping and development of this initial feasibility study. Work with this wider Stakeholder Group will also facilitate ongoing co-production of ambitions and plans for longer-term research into the impact of the HNT programme, subject, of course, to the conclusions from this initial feasibility study.

### Dissemination

We will disseminate findings using appropriate formats and print, online and social media for audiences including local governmental organisations, planners and developers, and local stakeholders. Findings will be published in peer-reviewed and practitioner publications. Presentations will be made at academic and practitioner conferences (e.g. national and international public health conferences). The findings will also be reported as briefing papers to commissioners, managers and the public.

## Discussion

The HNT programme provides a unique opportunity to examine a cross-section of new housing developments in England that are underpinned by healthy planning principles. This baseline and feasibility study has the potential to provide valuable information on the systems within which these new developments are operating, the expected outcomes of the programme and the processes through which outcomes may be influenced. Furthermore, this research is necessary to understand how longer-term health, wellbeing, behavioural and economic outcomes can be measured and monitored to generate evidence on the effectiveness of the HNT programme in a definitive longer-term study.

Research findings have the potential to influence local HNT stakeholders, including residents, by reinforcing awareness of healthy design principles and the importance of evaluating impact of their application on health and wellbeing. There is potential in the longer term for findings of this study to influence healthy planning processes, through adoption by local authorities, planners and developers of best practice, case studies and guidance to promote application of healthy design principles in other new housing developments. Developing a conceptualised system for complex interventions such as HNTs may be useful to guide similar programmes to build healthy urban environments in new and existing urban areas.

## Conclusions

Findings from this research will allow us to show to what extent this approach can be effective for developing and analysing causal pathways to health improvement through complex systems where outcomes may only manifest over longer time periods. Findings will also have potential value for planners, developers, policy-makers, funders and scientists, by contributing new outcome measures on which to base future investigations of impact, costs and benefits of the HNT programme. For example, we will generate an understanding of how primary and secondary outcomes for the overall HNT programme can be defined, and whether these outcomes can be measured using routinely available and locally held data and what local (non-routinely collected) data needs to be captured. We will also develop an understanding of the feasibility of evaluating the effectiveness of the HNT programme by matching HNT demonstrator sites to comparator sites retrospectively using routinely available and locally held data. This research will provide an understanding of the opportunities and barriers to conducting longer-term research and how these barriers may be overcome. Finally, this research will establish the feasibility of a definitive study to assess the long-term health impacts of HNTs

## Data Availability

Not applicable

## Abbreviations

CLD: Causal loop diagram
HNT: Healthy New Town
HNTEC: Healthy New Town Evaluation Collaborative
MSOA: Middle Layer Super Output Area
NHSE: National Health Service England
PHE: Public Health England
RG: Residents’ Group
